# VirtMRI: A Tool for Teaching MRI

**DOI:** 10.1007/s10916-023-02004-4

**Published:** 2023-10-25

**Authors:** Christian Tönnes, Christian Licht, Lothar R. Schad, Frank G. Zöllner

**Affiliations:** 1 Computer Assisted Clinical Medicine, Medical Faculty Mannheim, Heidelberg University, Mannheim, 68167 Germany; 2 Mannheim Institute for Intelligent Systems in Medicine, Medical Faculty Mannheim, Heidelberg University, Mannheim, 68167 Germany

**Keywords:** Magnetic resonance imaging MRI, Medical training/Educational tool, Free open source software FOSS

## Abstract

Magnetic resonance image formation is not trivial and remains a difficult subject for teaching. Therefore, we saw an urgent need to facilitate teaching by developing a practical and easily accessible MR image generator. Due to the increasing interest in X-nuclei MRI, sodium image generation is also offered. The tool is implemented as a web application that is compatible with all standard desktop browsers and is open source. The user interface focuses on the parameters needed for the creation and display of the resulting images. Available MR sequences range from the standard Spin Echo and Inversion Recovery over steady-state to conventional sodium and more advanced single and triple quantum sequences. Additionally, the user interface has parameters to alter the resolution, the noise, and the k-space sampling. Our software is free to use and specifically suited for teaching purposes.

## Introduction

Teaching (medical) students about MRI is a balancing act between quantum physics and understandable application. In our medical school, medical students are taught very compressed about the physics of MRI systems at the start of the first semester, and they have a short seminar where a table-top MRI device and a program for generating MRI images are used to show how sequences work and contrasts are generated. A second longer seminar with the same table-top MRI and program is in the fourth year. So far, we have used the software by Hackländer and Mertens [1] for our teaching. It is a Java program that enables students to test different sequences and the influence of various parameters. The software also supports noise addition, k-space manipulations, and motion artifacts. A disadvantage is that students can access it only during class and hence, we saw an urgent need to develop a tool that is remotely accessible. To the best of our knowledge, the only MRI image generator for teaching that is able to easily solve the accessibility problem published in the last decade is by Treceño-Fernández et al. [2]. This system is web-based and therefore, could be made accessible over the internet. It also allows students to test different sequences, set the parameters, add different types of noise, manipulate k-space, and use different B0 inhomogeneities. This tool performs all the calculations exclusively on the server, which leads to high server load and bandwidth usage. Therefore, it is suitable for a class setting using the local network, but probably does not scale well if large groups of students access the web page simultaneously over the internet. In addition to this, Treceño-Fernández et al. focus more on the usage of MRI devices and matched their workflow and user interface to those of real MRIs, while the tool presented here aims to demonstrate differences between sequences and the resulting images. Apart from these two MR image generators, there are a number of simulators published in recent years that run on the local computer [3–6]. Those simulators are mostly developed for researchers or physics and engineering students. For non-technical students like medical students installing programs or Java, using Matlab, simulators that only run on selected operating systems or complicated interfaces that need in-depth knowledge about Bloch equations or sequences make these simulators inaccessible. We present here a different approach to a web-based image generator that performs all computations on the client to eliminate the scaling problem and has a lean user interface.

## MRI generation

For our system, the main goal was to create a teaching tool that is compact, usable across many platforms, intuitive, and with minimal load on the web server.

### Requirements

The work for the server should be minimal, which is realized by performing all computations by the client’s device. This requires a system with low computational overhead so that users can run the tool on smartphones or tablets. Programming languages such as HTML, CSS, and JavaScript were used so that the program can be run on any platform with a modern browser. Therefore, the main objective was to make it independent of having a specific operating system, 3^rd^ party software or device. Hence, the following prioritization list has been derived to guide the development of the presented software:

1. low server load
2. remote accessibility
3. cross-platform
4. low resource usage
5. convenient and clear GUI
6. small file sizes

### Functions

MRI enables users to create different contrasts between tissues by exploiting different magnetization properties. Therefore, we included multiple sequences, and besides standard hydrogen (¹H), sodium (²³Na) imaging was also included.

Currently, the system supports six basic sequences for ¹H MRI, Spin Echo, Inversion Recovery, and more advanced sequences such as spoiled gradient echo.

The symbols used in the following equations are explained in Table 1

**Spin echo **[7, 8]

1 $$\begin{aligned} S \propto pd * e^{\frac{-TE}{T2}} * (1 - e^{\frac{-TR}{T1}}) \end{aligned}$$

**Inversion recovery **[9, 10]

2 $$\begin{aligned} S \propto pd * (1.0 - 2.0 * e^{\frac{-TI}{T1}} + e^{\frac{-TR}{TI}}) * e^{\frac{-TE}{T2}} \end{aligned}$$

**Spoiled gradient echo **[11, 12]

3 $$\begin{aligned} S \propto pd * \frac{(1-e^{\frac{-TR}{T1}})*sin(FA)}{1-e^{\frac{-TR}{T1}}*cos(FA)} * e^{\frac{-TE}{T2*}} \end{aligned}$$

Also included are three steady-state sequences. These are common sequences available on commercial MRI scanners and provide contrasts different from the previous three sequences.

**Balanced steady-state free precession (True FISP/FIESTA/Balanced FFE) **[13, 14]

4 $$\begin{aligned} S \propto pd * \frac{(1-e^{\frac{-TR}{T1}})* sin(FA)}{1 - (e^{\frac{-TR}{T1}}-e^{\frac{-TR}{T2}})*cos(FA) - e^{\frac{-TR}{T1}}*e^{\frac{-TR}{T2}} } * e^{\frac{-TE}{T2}} \end{aligned}$$

**Postexcitation refocused steady-state precession (FISP/GRASS, fast MPGR/FFE) **[14]

5 $$\begin{aligned} \begin{aligned} S \propto \;&pd * tan(\frac{FA}{2}) * e^{\frac{-TE}{T2*}} * \left( 1 - (e^{\frac{-TR}{T1}}-cos(FA))*f(TR,T1,T2,FA)\right) \\ f(TR,T1,T2,FA) =&\sqrt{\frac{1-e^{\frac{-2*TR}{T2}}}{ (1-e^{\frac{-TR}{T1}}*cos(FA))^2-e^{\frac{-2*TR}{T2}}*(e^{\frac{-TR}{T1}}-cos(FA))^2}} \end{aligned} \end{aligned}$$

**Preexcitation refocused steady-state precession (PSIF/SSFP/T2-FFE) **[14]

6 $$\begin{aligned} &S \propto \;pd * tan(\frac{FA}{2}) *e^{\frac{-TE}{T2}}\\&*\left( 1-(1-e^{\frac{-TR}{T1}}*cos(FA))* f(TR,T1,T2,FA)\right) \end{aligned}$$

For ^23^Na imaging, we implemented the signal equation for ^23^Na, enabling the creation of conventional ^23^Na MR images. However, sodium is a quadrupole in its nature and thus, exhibits multi-quantum properties. Under certain conditions, one can observe besides the Single Quantum (SQ) also Triple Quantum (TQ) signal, which could provide a richer tissue sodium characterization. Hence, we implemented CRISTINA [15] so we can generate three ^23^Na images: conventional, single-, and triple-quantum. The single- and triple-quantum images can be further used to calculate the ratio of triple- to single-quantum signal.

^**23**^**Na signal **[16]

7 $$\begin{aligned} &S \propto \;(na_{vol}-vol)* mm * (1-e^{\frac{-TR}{T1}}) \\&* (0.6*e^{\frac{-TE}{T2f}} + 0.4*e^{\frac{-TE}{T2s}}) + vol*na_{mm} \\&* (1-e^{\frac{-TR}{T1}})*e^{\frac{-TE}{T2fr}} \end{aligned}$$

**Single quantum spin echo **[15]

8 $$\begin{aligned} S_{sq} \propto \frac{1}{|TEs|} \sum _{TE \in TEs}{mm * ( e^{\frac{-TE+\tau _1}{T2s}} + e^{\frac{-TE+\tau _1}{T2f}} ) * sin(FA) } \end{aligned}$$

**Triple quantum spin echo **[15]

9 $$\begin{aligned} &S_{tq} \propto \frac{1}{|TEs|} \sum _{TE \in TEs} mm * (e^{\frac{-TE}{T2s}} - e^{\frac{-TE}{T2f}}) \\&* (e^{\frac{-\tau _1}{T2s}}-e^{\frac{-\tau _1}{T2f}}) * e^{\frac{-\tau _2}{T2s}} * sin(FA)^5 \end{aligned}$$

**TQ/SQ spin echo**

10 $$\begin{aligned} S \propto \frac{S_{tq}}{S_{sq}} \end{aligned}$$

For all these functions, the user can change the used parameters. The parameters are mostly the echo time, the repetition time, or the flip angle. For most sequences, we give an estimate of the acquisition time that a real MRI device would need.

**Table 1:** Explanation for the symbols used in the signal equations

	Symbol	Description	Comment
	S	Measured signal strength	proportional to real signal
Tissue Parameters	pd	Proton Density	only ^1^H imaging
	T1	T1 Relaxation Time	
	T2	T2 Relaxation Time	only ^1^H imaging
	T2*	T2* Relaxation Time	only ^1^H imaging
	T2f	T2 Time, fast component	only ^23^Na imaging
	T2s	T2 Time, slow component	only ^23^Na imaging
	mm	sodium concentration	in mmol/ml
	vol	fraction of extracellular sodium	
	$na_{vol}$	voxel fraction containing sodium	fixed to 0.7
	$na_{mm}$	sodium in water	fixed to 140mmol/ml
	T2fr	T2 Time for free sodium	fixed to 60ms
Sequence Parameters	TE	Echo Time	set by user in milliseconds
	TR	Repetition Time	set by user in milliseconds
	TI	Inversion Time	set by user in milliseconds
	FA	Flip Angle	set by user in degree
	$\tau _1$	Time between 1^st^ and 2^nd^ RF pulse	set by user in milliseconds
	$\tau _2$	Time between 2^nd^ and 3^rd^ RF pulse	set by user in milliseconds

Furthermore, subsampling with different interpolation modes, Gaussian noise, simple k-space manipulation, and 2D or 3D Fourier transform is supported.

For undersampling of the k-space we give a choice of three schemes: Random, density-adapted Pseudo-Random, and Regularly spaced. Random means an arbitrary decision to include or discard a voxel in k-space, yielding non-Cartesian k-space trajectories. The other two schemes retain or discard complete phase-encoding lines in k-space, representing Cartesian trajectories. Regularly spaced means for a 50% sampling fraction $f_s$ that every second line is measured, for 33% every third. The condition for measuring a line *y* is shown in Eq. 11. If the condition for *measure*(*y*) is true, then the line *y* is measured; otherwise it will be dropped. The line numbering *y* starts at 1.

11 $$\begin{aligned} measure(y) = \left\{ \begin{array}{llr} \frac{\textrm{cei}l\left( y\, \cdot f_{s} \right) }{ f_{s}}-y &{}< 1 &{} f_{s}<0.5 \\ \frac{\textrm{cei}\left( y\,(1-f_{s}) \right) }{ (1-f_{s})}-y &{}\ge 1 &{} f_{s}\ge 0.5 \\ \end{array} \right. \end{aligned}$$

A commonly used sampling scheme is the density-adapted pseudo-random sampling, which keeps the full k-space center, and the probability to drop a line increases with the distance from the center. This is a common sampling scheme for compressed sensing [17]. We always keep a fraction $f_{in}$ 10% in the center of the complete k-space. Then a random number is generated at each line and compared to a linearly decreasing threshold. The parameters for this threshold are chosen so that the resulting sampling rate is the selected sampling rate. The calculation for these parameters is shown in Eq. 12. In these equations, $Dim_y$ is the total number of *y* lines.

12 $$\begin{aligned} \begin{array}{ll} b &{}= \left\{ \begin{array}{lr} \frac{f_s - f_{in}}{0.5\,(1-f_{in})} - 1 &{} f_s<0.5\\ 2 - \frac{f_s - f_{in}}{0.5\,(1-f_{in})} &{} f_s\ge 0.5 \end{array}\right. \\ a &{}= \frac{b}{1-f_{in}} \\ \Psi (y) &{}= \left\{ \begin{array}{lr} 2*y/Dim_y &{} y / Dim_y < 0.5\\ 2*(1-y/Dim_y) &{} y / Dim_y \ge 0.5\\ \end{array}\right. \\ measure(y) &{}= r_{random} \le -a\,(\Psi (y)-f_{in})+b \end{array} \end{aligned}$$

### Architecture

The architecture can be viewed on two levels. There is a server-client architecture to deliver the web app to the browser. Here we use static files which can be served by every standard web server. This project is based on the SimpleHttpServer included in Python 3.

The web page uses the Model-View-Controller pattern and offloads the computation to a worker thread. It only connects to the server to load a data set. After that, all computation and data handling is performed within the browser. The view is written in HTML and CSS using the CSS files from the Bootstrap project. Some responsive behavior, e.g. calculating the needed time for a scan, is calculated in JavaScript. The controller uses JavaScript and most of the computation is written in both JavaScript and c/WebASM. An exception is the FFT, where we use the KissFFT project, which is only written in c and then compiled with emscripten to WebASM. This is done to speed up the computations. We purposely did not write everything in c/WebASM so that an interested user can simply open the web developer tools and follow the computation with the built-in debugger. The WebASM version of the image creation process has a faster runtime and is therefore set as the default computation backend.

### Data sets

Each data set consists of multiple 3D arrays for the different parameter maps. For 1H MRI that includes: T1, T2, T2*, and proton density. For ^23^Na, the parameters are T1, T2 fast, T2 slow, sodium density, and extracellular volume fraction. Every array has a size of 256x256x256 voxels and was generated using published head phantoms [18–21]. The data sets generated using Aubert-Broche et al. [18, 19] and Holmes et al. [20] are available for 3T and 1T ^1^H and 3T ^23^Na MRI and the data set generated from Alfano et al. [21] is 1T and 1.5T ^1^H MRI. The phantoms we used consisted of segmentation masks for different tissue types. We used these to generate the parameter maps by simply inserting the values for each parameter found in the literature (Tables 2). These maps were then resampled to 256x256x256 voxels.

**Table 2:** Parameters used for 1.5T ^1^H, 3T ^1^H and 3T ^23^Na images. Parameter names are in analogy to [18]. *: Parameters were not found, approximated with values for fat/muscle

Parameters for 1.5T ^1^H [19]					
Tissue	T1 [ms]	T2 [ms]	T2* [ms]	PD	
Background	0	0	0	0	
CSF	2569.0	329	58	1	
Grey Matter	833	83	69	0.86	
White Matter	500	70	61	0.77	
Fat	350.0	70.0	58	1	
Muscle	900.0	47	30	1	
Muscle / Skin	569.0	329	58	1	
Skull	0	0	0	0	
Vessels	2569.0	329	0	1	
Dura Mater	2569.0	329	58	1	
Bone Marrow	500.0	70	61	0.77	

Parameters for 3T ^1^H [22–24]					
Tissue	T1 [ms]	T2 [ms]	T2* [ms]	PD	
Background	0	0	0	0	
CSF	4163.0	329	58	1	
Grey Matter	1445	83	66	0.86	
White Matter	791	75	53.2	0.77	
Fat	346	68	58	1	
Muscle	1420	44	30	1	
Muscle / Skin	371.0	133	58	1	
Skull	0	0	0	0	
Vessels	1984.4	275.0	0	1	
Dura Mater	2569.0	329	58	1	
Bone Marrow	365	133	61	0.77	

Parameters for 3T ^23^Na [25, 26]					
Tissue	T1 [ms]	T2 slow [ms]	T2 fast [ms]	Extracellular fraction	Sodium [mmol]
Background	0	0	0	0	0
CSF	50	60	60	1	140
Grey Matter	30	60	2	0.21	55
White Matter	30	60	2	0.17	45
Fat	10	50	4	0.2	0
Muscle	25.2	30	2	0.2	20
Muscle / Skin*	25.2	30	2	0.2	20
Skull	0	0	0	0	0
Vessels	38.4	20	3	1	150
Dura Mater*	10	50	4	0.2	0
Bone Marrow*	10	50	4	0.2	0

### Image generation pipeline

**Fig. 1:**
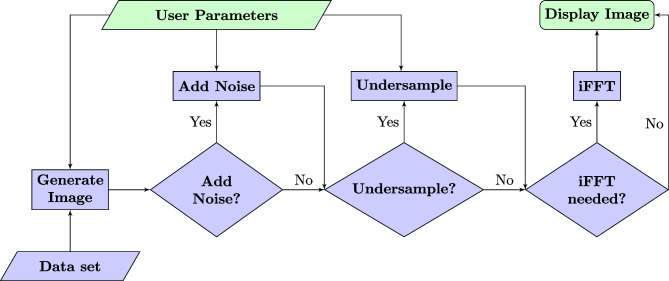
Image generation pipeline. The green boxes are part of the front end and the blue ones are in the back end

Our generation process (Fig. 1) is quite straightforward. MR images are computed in the image domain using the Eqs. 1 to 10 for every voxel and followed by a Fourier transform to calculate the k-space. For added noise, random numbers, chosen from a Gaussian distribution, are added to each value in the k-space and if undersampling is activated, the k-space is filtered using the selected sampling scheme to remove a configurable percentage of the total lines prior to the inverse Fourier transformation. If the k-space was modified, an inverse Fourier transform is used to compute the final image to be displayed.

### GUI

**Fig. 2:**
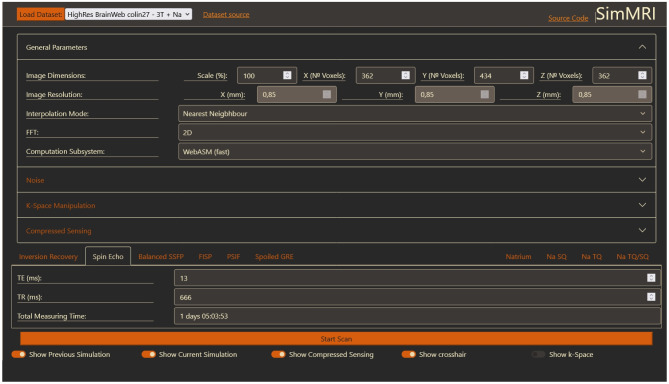
The GUI after loading a data set. General parameters are stated at the top and sequence specific parameters are found at the bottom

The user interface is written using HTML and CSS. The base CSS files are from the bootstrap project (version 5) [27], a toolkit to build web frontends. The dark Gruvbox [28] scheme was chosen for the color theme. When the user opens the web page, they first have to choose a data set. After loading the data set, the input fields for general parameters and sequences become visible (Fig. 2). The link "Dataset source" next to the drop-down box always links to the webpage of the selected data set, where the input files for each data set can be downloaded. An MRI sequence can be selected by clicking on the corresponding tab, which also visualizes the specific parameters for this sequence. The parameters for each sequence are independent, e.g. changing the Echo Time in Inversion Recovery does not change the Echo Time for Spin Echo. Only the selected and visible parameters are used for a sequence, except for the ’^23^Na TQ/SQ’ sequence, which uses the parameters of the ’^23^Na SQ’ and ’^23^Na TQ’ tabs. The field ’Total Measuring Time’ provides an approximation of the time required to conduct the selected MRI experiment.

**Fig. 3:**
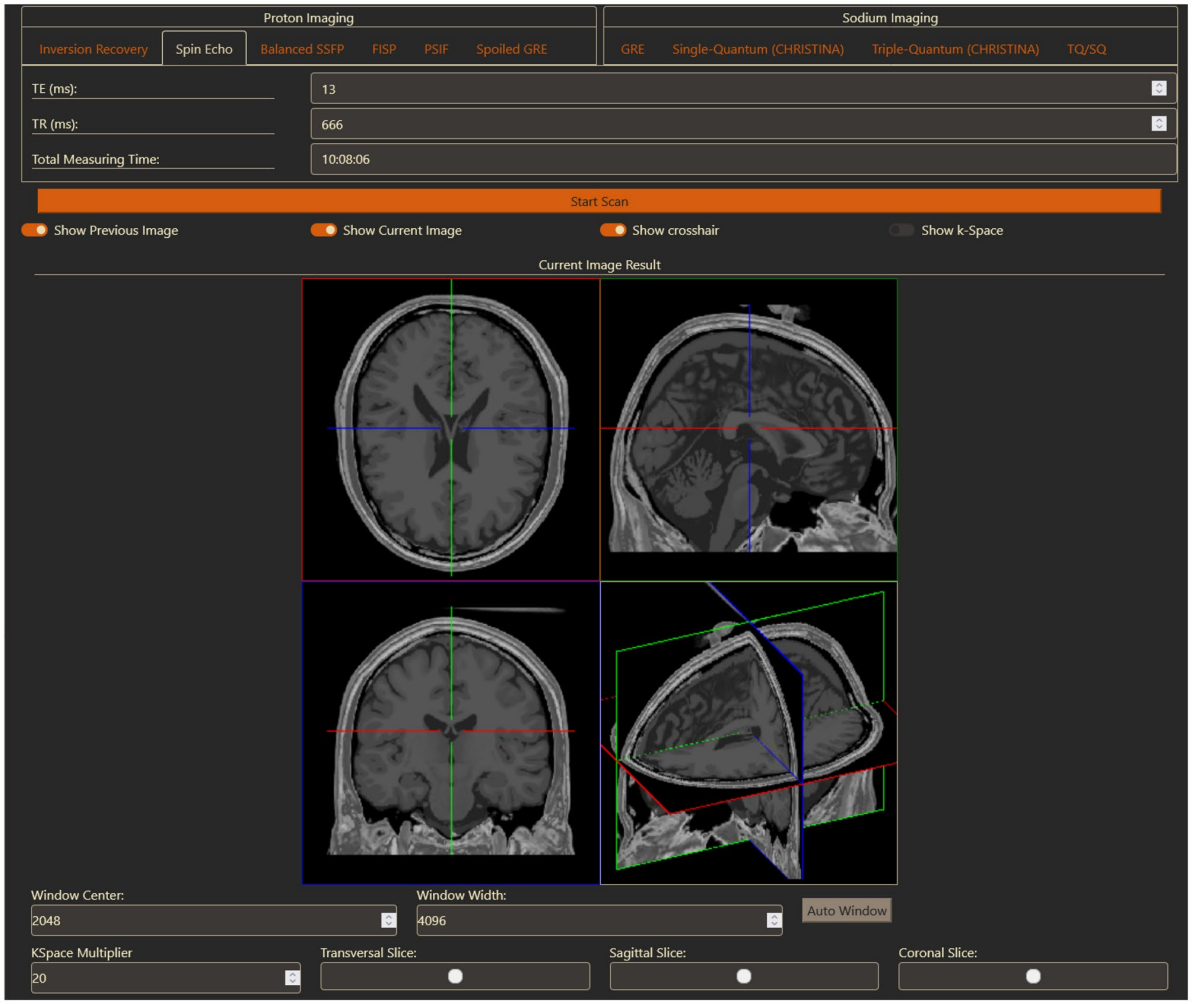
Resulting signal computation for a Spin Echo acquisition based on Eq. 1. The Viewer shows a slice from the transversal, sagittal, and coronal planes and then these three slices in a 3D view. Control panels to manipulate and navigate through the images are found beneath the images

The general parameters are in an accordion menu and can be expanded or collapsed as needed. In the screenshot, the menu "General Parameters" is expanded and the menus for noise and compressed sensing are collapsed. Collapsed menus have a different font color to emphasize that they can be expanded. We chose this to signify that all general parameters are always used for image generation, except for the sequence parameters, only the ones on the currently active and visible tab are used. However, displaying all parameters at once creates an overloaded interface, so the user has the option to collapse them. The button with the label "Start Scan" starts the generation process. The computed images will be displayed below (Fig. 3). The toggle buttons allow the user to select which images should be displayed and to show or hide the respective k-space.

Every image is displayed in a four-panel view. The top left corner contains a transversal, the top right a sagittal, and the bottom left a coronal slice. The bottom right quarter is either the k-space or a 3D view of the current slices, which also allows for rotation of the view.

**Fig. 4:**
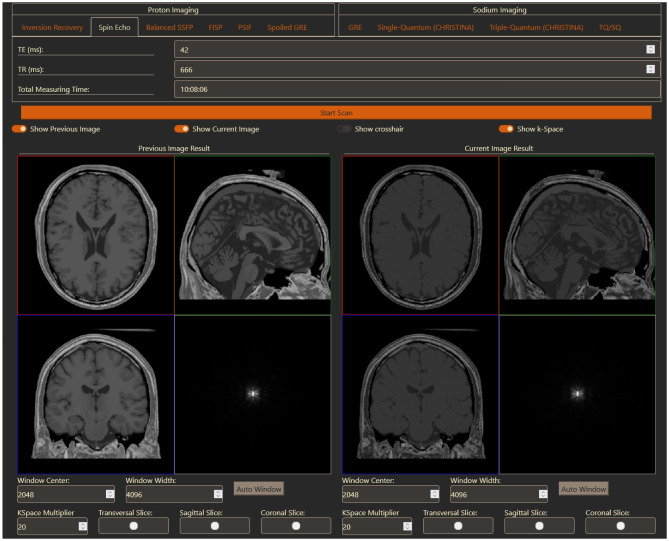
Generation of a second Spin Echo image. On the left-hand side is the previous Spin Echo image from Fig. 3 and on the right side the new Spin Echo image is shown. The crosshair has been turned off and the 3D view replaced with the k-space

After generating a second image, the user can now decide to view both of them next to each other (Fig. 4) or only one of them. Figure 4 shows a comparison of two Spin Echo images with different TE values. Additionally, the crosshair has been hidden and the 3D view is replaced with the respective k-spaces.

The user can interact with the other sections of the image by holding the mouse button and moving it, which changes the center and width of the window. The slice can be changed with the mouse wheel. Both the windowing and the slice can also be selected using the input fields below the image. When a 23Na is created, the selection fields for the windowing are replaced by a color bar. Slice selection for the image and the k-space is partly synchronized. Scrolling through one image also scrolls through the other visible images, enabling users to compare images with different acquisition parameters and sequences. Slice selection using the input boxes below the image is not synchronized. This allows a user to set every image to a different slice. In that case, the scrolling is still synchronized, but the offset between the slices is kept until one image is at the first or last slice.

To generate an image with a lower resolution, the value of the scale field has to be changed. The interpolation mode then decides how to calculate the voxel value. Possible options are to use the nearest neighbor or to average over all voxels in the data set that would be within the virtual image voxel.

## Results

We will focus on the impact of changing the general parameters, resolution, interpolation mode, 3D vs. 2D FFT, computational subsystem, and noise (Table 3). For all parameters, the JavaScript version was much slower than WebASM. Chrome was always slower than Firefox when using WebASM, but Chrome was faster most of the time when using the JavaScript version. Generating an image using only the nearest point of the data set to the center of a voxel is faster than averaging over all data points inside the voxel. Reducing the size of the generated image also reduces the computation time, since the Fourier transformations are quicker and they take up a big share of the total computation time. When noise is added to the k-space, an additional inverse Fourier transform is required to obtain the noisy image, which, as expected, increases the running time.

**Table 3:** Runtimes for several parameter combinations using a Spin Echo sequence (TE: 23, TR: 666). All parameter combinations are computed with the slow JavaScript and the faster WebASM version. The runtimes are averaged over 10 runs, on a PC with Intel i5-6500 CPU and 64GB RAM. Maximum RAM used by the Browsers: Firefox 1.8GB, Chrome 1.9GB

Noise	XxYxZ	Interpolation	FFT	Compute	Runtime	
				Subsystem	Firefox [s]	Chrome [s]
No	256x256x256	Nearest	3D	JavaScript	$6.85 \pm 0.70$	$\varvec{5.85 \pm 0.12}$
				WebASM	$\varvec{2.98 \pm 0.12}$	$4.16 \pm 0.26$
			2D	JavaScript	$6.57 \pm 0.69$	$\varvec{6.03 \pm 0.81}$
				WebASM	$\varvec{3.13 \pm 0.49}$	$4.19 \pm 0.46$
		Average	3D	JavaScript	$7.78 \pm 0.81$	$\varvec{7.50 \pm 1.05}$
				WebASM	$\varvec{3.08 \pm 0.17}$	$4.29 \pm 0.44$
			2D	JavaScript	$7.66 \pm 0.54$	$\varvec{7.51 \pm 1.00}$
				WebASM	$\varvec{3.16 \pm 0.22}$	$4.31 \pm 0.48$
No	256x256x64	Nearest	3D	JavaScript	$1.66 \pm 0.14$	$\varvec{1.52 \pm 0.19}$
				WebASM	$\varvec{0.77 \pm 0.05}$	$1.07 \pm 0.13$
			2D	JavaScript	$1.65 \pm 0.10$	$\varvec{1.54 \pm 0.20}$
				WebASM	$\varvec{0.74 \pm 0.04}$	$1.06 \pm 0.09$
		Average	3D	JavaScript	$3.83 \pm 0.23$	$\varvec{2.91 \pm 0.33}$
				WebASM	$\varvec{1.51 \pm 0.09}$	$2.34 \pm 0.25$
			2D	JavaScript	$4.09 \pm 0.44$	$\varvec{2.94 \pm 0.40}$
				WebASM	$\varvec{1.68 \pm 0.28}$	$2.36 \pm 0.27$
Yes	256x256x256	Nearest	2D	JavaScript	$11.38 \pm 2.07$	$\varvec{10.40 \pm 1.37}$
				WebASM	$\varvec{4.65 \pm 0.51}$	$7.12 \pm 0.97$
Yes		Average	2D	JavaScript	$\varvec{11.99 \pm 1.57}$	$12.20 \pm 1.83$
				WebASM	$\varvec{4.58 \pm 0.51}$	$7.48 \pm 1.13$
	256x256x64	Nearest	2D	JavaScript	$\varvec{2.72 \pm 0.31}$	$2.77 \pm 0.52$
				WebASM	$\varvec{1.20 \pm 0.13}$	$1.86 \pm 0.29$
		Average	2D	JavaScript	$4.92 \pm 0.68$	$\varvec{4.26 \pm 0.84}$
				WebASM	$\varvec{1.94 \pm 0.20}$	$3.19 \pm 0.51$

**Fig. 5:**
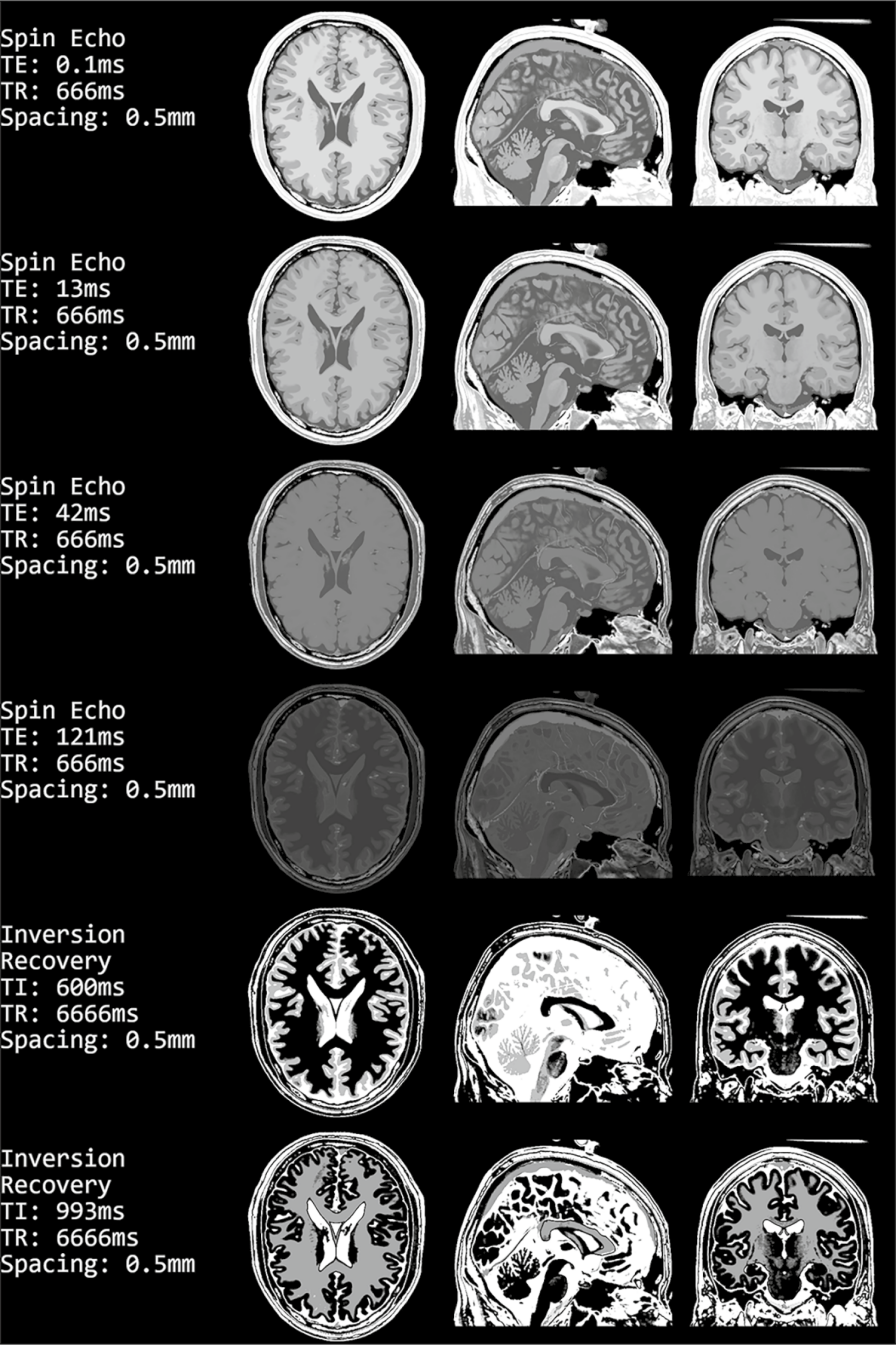
Generated images using the Spin Echo sequence (Eq. 1) with different Echo Times and Inversion Recovery sequence (Eq. 2) with different inversion times. In all rows, the shown slices are the middle slice in the transversal, sagittal, and coronal planes

Spin Echo and Inversion Recovery images generated with the here proposed software are shown in Fig. 5. The first four rows show generated images using Spin Echo and different echo time, ($TE_1=0.1ms$,$TE_2=13ms$,$TE_3=42ms$,$TE_4=121ms$). Furthermore, two Inversion Recovery images are shown with different inversion times chosen to suppress White Matter ($TI_1=600ms$) and Grey Matter ($TI_2=993ms$).

**Fig. 6:**
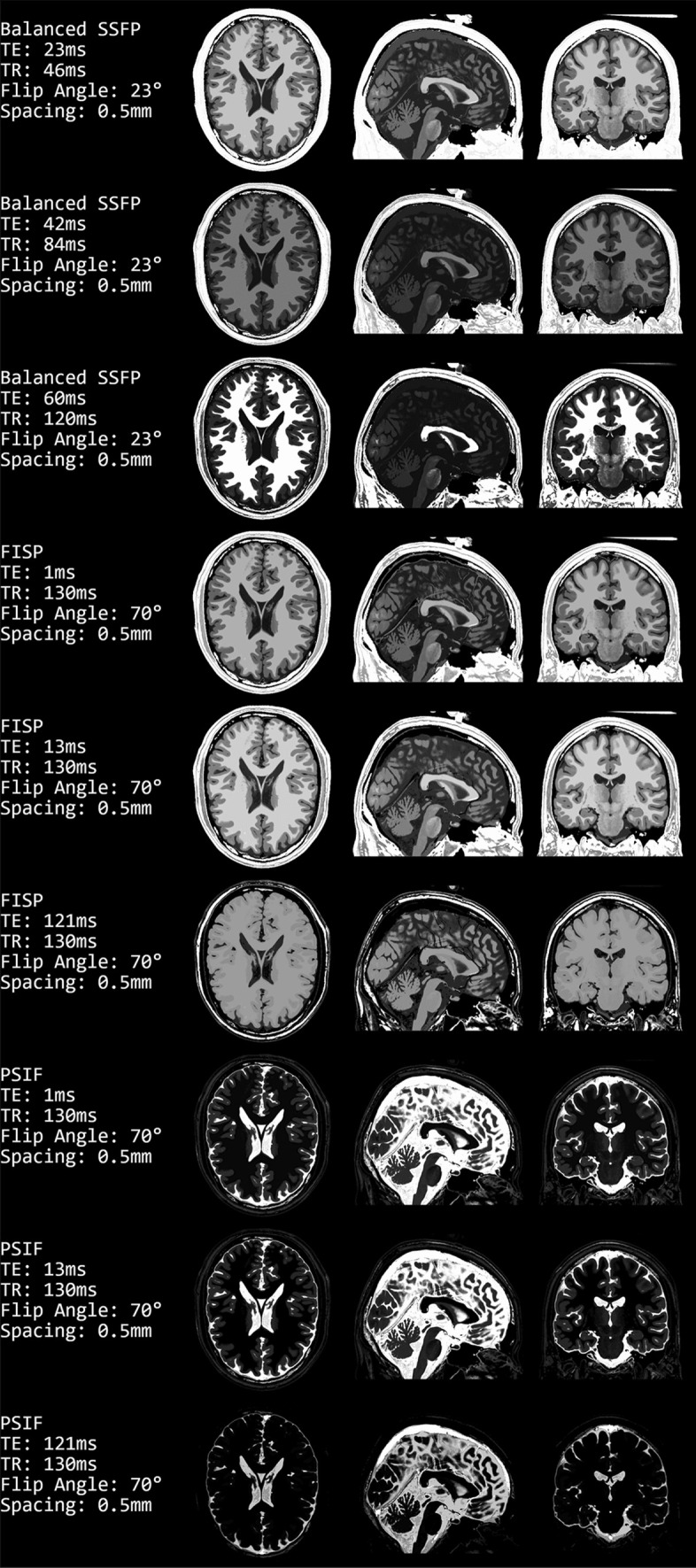
Images computed using the steady-state sequences balanced SSFP (Eq. 4), FISP (Eq. 5), and PSIF (Eq. 6)

The steady-state sequences are shown in Fig. 6. Similar to the previous figure, different parameters are used in each row and the same three slices of the head are shown.

**Fig. 7:**
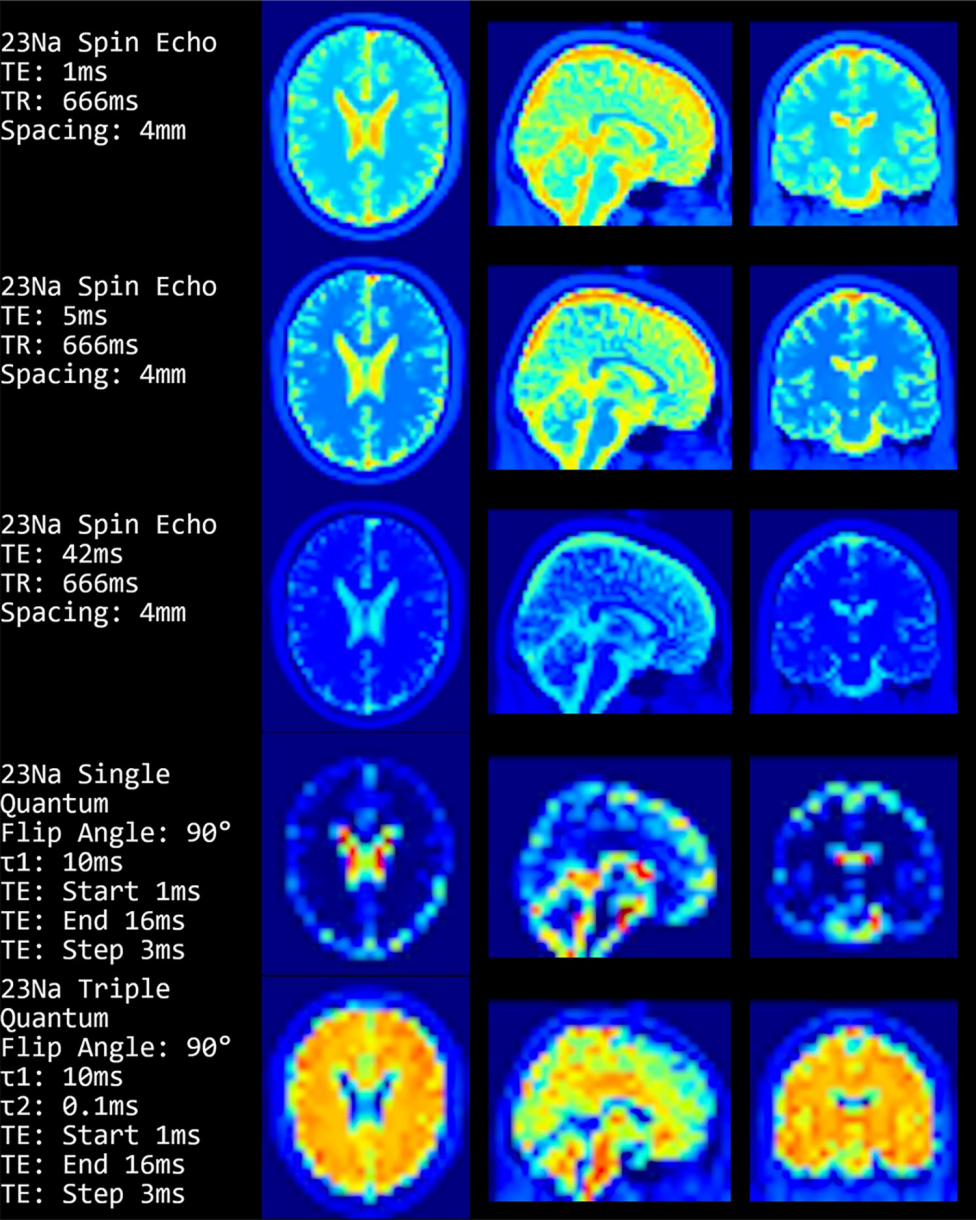
Images were computed using the equations for the sodium sequences. The top three rows show ^23^Na Spin Echo (Eq. 7), then follows a single quantum (Eq. 8) and a triple quantum (Eq. 9) image. The voxel size and spacing for the single/triple quantum images is 16mm

Figure 7 shows the Spin Echo sodium sequences in addition to single and triple quantum imaging. These images are generated with a reduced resolution to better resemble state-of-the-art for sodium imaging in reality. The Spin Echo images are downsampled to an isotropic voxel size of 4mm and the single/triple quantum images have a voxel size of 16mm.

## Discussion

In summary, we have presented a web-based image generator designed for teaching. The software allows the generation of signals based on different sequences and the demonstration of the influence of the parameters. It supports a vast amount of sequences ranging from standard proton Spin Echo to more advanced sodium sequences. Further, the system contains the options to add noise, change image resolution, and k-space undersampling with different strategies. Secondly, a user-friendly interface was developed that eases usage. Additionally, the software can run on a wide range of devices, which is attributed to the fact, that the software was developed as a web-based application. Lastly, the server management and costs are reduced since we have only static files. No computation is necessary on the server, and static files can be distributed to many clients without much effort. Deploying to a new server is also simple, just copy the "wwwroot" folder from the GitHub repository [29], this contains all the required source and data files.

Our tool started as a small piece of software, but with added functions, it increased in size and computational cost. While still able to be used on smartphones, a user has to wait for some time until the computation process is finished. One solution to reduce computational cost would be to detect mobile devices like a smartphone and then provide a reduced version of the web page, e.g. only allowing nearest-neighbor interpolation and 2D Fourier transformation. Another solution, which we implemented already, is to code the computationally intense algorithms in c and then compile them to WebASM. This makes the computation process less transparent because interested users would not be able to simply open the web debugger (which can be accessed in most major browsers by pressing F12) and look at how the program runs inside their browser. On the other hand, we see that the WebASM version only takes  50% of the time required by the JS version to calculate an image. This will allow a user to choose between the slow, but debuggable, JavaScript and the fast, oblique WebASM version.

Future work will focus on including parallel imaging and compressed sensing. Both are implemented in modern MRI devices and are quite interesting.

The added noise and image artifacts are quite basic. So far, the user can only select Gaussian noise. A possible artifact we could add without much hassle is B0 homogeneity by extending the image creation process with the inclusion of a static homogeneity map. The movement of the patient would be somewhat more difficult. To include a single and fast movement of the complete patient, the interpolation grid could be shifted and rotated during the computation. This would require computing the images and k-spaces twice, and then merging these k-spaces so that the points captured before and after the motion are from the corresponding k-space. While this is not a perfect representation of patient movement, it should be a usable approximation and starting point for more complex movements. The flexible interpolation grid required for the proposed patient motion artifacts could also be used for other purposes, such as changing the orientation of the slices. Setting the slice orientation could be done by simply changing values for the rotation in several input boxes. But we think this would not be intuitive and a better approach is a 3D view, similar to what Treceño-Fernández et al. implemented.

Other tools focus on having a GUI that resembles a real MRI machine. However, we focused on convenience in regard to usage and accessibility, which was the reason to neglect the implementation of a scanner related interface. The workflow for acquiring images on a real MRI is beyond the scope of this software.

In conclusion, we have presented a web-based image generator for a wide range of MR sequences that is scalable, cross-platform, and freely available.
